# TGFβ_1_ priming enhances CXCR3‐mediated mesenchymal stromal cell engraftment to the liver and enhances anti‐inflammatory efficacy

**DOI:** 10.1111/jcmm.17698

**Published:** 2023-02-23

**Authors:** Abhilok Garg, Sheeba Khan, N. Luu, Davies J. Nicholas, Victoria Day, Andrew L. King, Janine Fear, Patricia F. Lalor, Philip N. Newsome

**Affiliations:** ^1^ National Institute for Health Research Birmingham Biomedical Research Centre, University Hospitals Birmingham NHS Foundation Trust University of Birmingham Birmingham UK; ^2^ Centre for Liver & Gastrointestinal Research, Institute of Immunology and Immunotherapy University of Birmingham Birmingham UK; ^3^ Liver Unit University Hospitals Birmingham NHS Foundation Trust Birmingham UK

**Keywords:** homing, immunomodulation, macrophages

## Abstract

The immunomodulatory characteristics of mesenchymal stromal cells (MSC) confers them with potential therapeutic value in the treatment of inflammatory/immune‐mediated conditions. Previous studies have reported only modest beneficial effects in murine models of liver injury. In our study we explored the role of MSC priming to enhance their effectiveness. Herein we demonstrate that stimulation of human MSC with cytokine TGβ_1_ enhances their homing and engraftment to human and murine hepatic sinusoidal endothelium in vivo and in vitro, which was mediated by increased expression of CXCR3. Alongside improved hepatic homing there was also greater reduction in liver inflammation and necrosis, with no adverse effects, in the CCL_4_ murine model of liver injury treated with primed MSC. Priming of MSCs with TGFβ_1_ is a novel strategy to improve the anti‐inflammatory efficacy of MSCs.

## 1 INTRODUCTION

Mesenchymal stromal cells (MSC) represent a promising therapeutic approach in many conditions, including inflammatory liver disease and graft versus host disease,^1^ as a consequence of their potent immunomodulatory properties.^2^ However, their efficacy in rodent and human models of liver injury has been variable, with some studies demonstrating benefit from MSC infusions^3^, ^4^, ^5^ whilst others report that infusion of conditioned medium from MSC cultures was sufficient to confer efficacy.^6^ Moreover, the mechanism of action by which MSC exert their effects in models of liver damage is poorly delineated with reports suggesting they may be mediated by a reduction in oxidative stress^3^ and/or reduced lymphocytic ingress to the injured liver with a secretome analysis suggesting this latter effect may be chemokine dependent.^6^

Whilst others have suggested that a component of MSC action may occur remotely without requirement for homing to the injured organ,^7^, ^8^ the relative lack of efficacy of MSC in models of liver injury has been attributed to low levels of MSC engraftment in the damaged liver. Using flow‐based assays we and other groups have demonstrated that β1 integrin and CD44 are involved in the firm adhesion of MSC to hepatic sinusoidal and human umbilical endothelium.^9^, ^10^ Notably, chemokine receptors did not appear to contribute significantly to human MSC recruitment,^11^ which was unexpected considering chemokine receptors play a significant role in leukocyte recruitment.^12^ Moreover, studies using murine MSC adhesion to murine aortic endothelium^13^ suggest a functional role of chemokine receptors in the firm adhesion, crawling and transmigration of MSC, although expression of chemokine receptors on human MSC such as CCR4 and CXCR3^14^ may be modest and different to murine cells. This variation in functional chemokine receptor profiles of MSC in reports from various groups^15^, ^16^, ^17^ has proven problematic in understanding the role of chemokine receptors in MSC homing and function. However, we have demonstrated that MSC detachment from tissue culture plastic can markedly affect expression of chemokine receptors, which may contribute to the variation in expression and function of MSC reported in the published literature,^18^ and also impact upon subsequent targeting in tissue. To mitigate for this, cell surface glycans on MSC have been chemically engineered into an E‐selectin binding motif in order to encourage engraftment to endothelium that expresses high levels of E‐selectin.^19^ Similarly pre‐loading of therapeutic MSC with paramagnetic nanoparticles has been utilized to allow specificity of delivery^20^; however, these methods of enhancing MSC migration are unlikely to be acceptable for clinical practice for logistical, safety and cost reasons.

Therefore, we explored the consequences of cytokine stimulation of MSC upon their hepatic engraftment and efficacy. We used cytokines known to increase inflammatory cell ingress and that are elevated in liver disease such as TNFα, IFNγ, TGFβ_1_, LPS, IL1β, IL4, IL6, IL8 and IL10.^21^, ^22^, ^23^, ^24^ Importantly, MSC have been reported to have receptors for these cytokines including TNFRI and IIR,^25^ IFNγR, TLR4,^26^ IL‐1R, IL‐4R, IL‐6R,^27^ IL8R (CXCR1)^25^ and IL10R.^28^ Herein we report that pre‐stimulation of clinically relevant human MSC with TGFβ_1_ enhances their binding/engraftment to hepatic sinusoidal endothelium ex vivo and in vivo in a CXCR3‐dependent manner and results in greater potency to reduce liver damage in an acute model.

## 2 MATERIALS AND METHODS

### 2.1 Human liver tissue and cell culture

Human liver tissue used in this study was obtained from patients at the Queen Elizabeth Hospital Birmingham, UK. Normal tissue was surplus to transplantation requirements or from tumour margin samples and diseased tissue was also obtained during transplantation for end‐stage disease (Primary Biliary Cirrhosis [PBC], Primary Sclerosing Cholangitis [PSC], Autoimmune hepatitis [AIH], Non‐alcoholic steatohepatitis^29^ and Alcoholic Liver Disease [ALD]). All samples were collected with local research ethics committee approval (reference number 06/Q2702/61) and informed, written patient consent. Freshly collected liver tissue was either snap frozen and sectioned to 10 μm for Stamper Woodruff adhesion assays or used for the isolation of hepatic sinusoidal endothelial cells (HSEC), biliary epithelial cells and hepatic myofibroblasts as previously described.^30^ Where indicated, cultured primary cells were treated with 10 ng/mL TNFα and IFNγ (both Peprotech) for 24 h prior to use in adhesion assays.

Human MSC from healthy donors were purchased from Lonza Group Ltd, (MSC: Lonza Poietics®) which are cryopreserved at Passage 2 and conform to International society of cellular therapy (ISCT) standards for surface marker expression (CD73^+^, CD90^+^, CD29^+^, CD105^+^, CD166^+^ and CD44^+^, CD14^−^, CD19^−^, CD34^−^, CD45^−^ and HLA DR^−^) and trilineage differentiation (Osteogenic, chondrogenic and adipogenic). Cells were cultured in human MSC Growth Medium (hGM) according to manufacturer's instructions and they were fully phenotypically characterized as we have described previously.^9^ Where indicated, MSC were stimulated with predetermined optimal concentrations of cytokines (TGFβ_1_, 5 ng/mL, IL‐4 10 ng/mL or IL‐10 50 ng/mL, all from Peprotech) or media alone, for 10 min to 24 h in hGM at 37°C.

### 2.2 Adhesion and migration assays

Adhesion of MSC to cultured cell monolayers, human liver tissue sections or mouse liver sections (control and CCl_4_ treated) was assessed using a modified Stamper Woodruff static adhesion assay. To assess migration of control or TGFβ_1_‐stimulated MSC we used a modified 48‐well Boyden chamber as previously described.^31^

### 2.3 Hepatic engraftment of MSC

All animal procedures were conducted in accordance with UK laws with the approval of the Home Office and local ethics committees (PPL 40/3201). Carbon tetrachloride (CCl_4_; Sigma Aldrich) diluted 1/4 in mineral oil (Sigma) was administered by intraperitoneal injections (1 mL/kg, twice weekly for 8 weeks or acutely as a single injection) into 9‐week‐old C57Bl/6 wild type male mice. Where indicated, MSC were pre‐incubated with blocking antibodies raised against chemokine receptors (anti human CXCR3, CCR5 or CXCR4 at 20 μg/mL, all from R+D systems) for 15 min at 37°C, washed and re‐suspended in PBS 0.1% BSA. To study engraftment of MSC into liver and non‐hepatic organs, MSC (control or 5 ng/mL TGFβ_1_‐stimulated) were labelled with Direct red (DiR 5 μM; Invitrogen) or CFSE according to manufacturer's instructions. Cells 1 × 10^6^ were either injected into the hepatic portal or tail vein of mice that had been acutely injured with CCl_4_ (1 mL/kg IP, 72 h). Organs were harvested 72 h later and imaged using an IVIS Spectrum Imaging System (Perkin Elmer). Fluorescent and photographic images of individual organs were analysed using Living Image software. Full details of all experimental protocols are available in Appendix S1.

### 2.4 Statistical analysis

Statistical analysis was performed by Student's *t* test or ANOVA using Prism software. Data are expressed as mean with standard errors. A value of *p* < 0.05 was considered significant.

## 3 RESULTS

### 3.1 MSC infusions reduce injury in the acute carbon tetrachloride model

**FIGURE 1:**
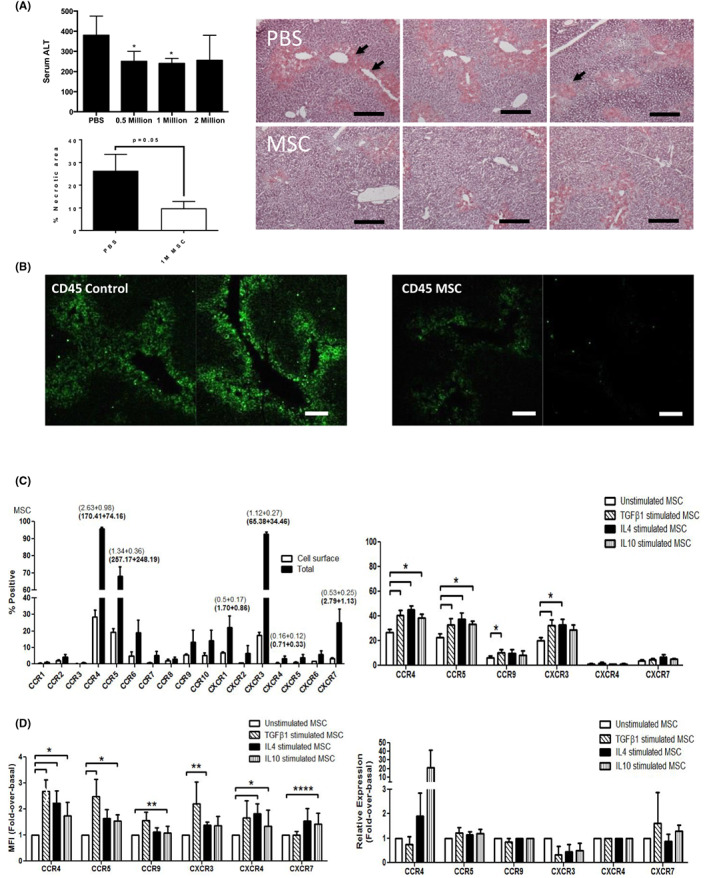
Efficacy of mesenchymal stromal cell (MSC) in acute liver injury and the impact of cytokine stimulation on chemokine expression. The effect of infusion of increasing doses of MSC was studied in mice with acute CCl_4_ injury. (A) Serum ALT levels (top left), and hepatic necrosis (bottom left and representative images on right) in mice treated with indicated doses of MSC or carrier (PBS). Representative haematoxylin and eosine (H&E) images are shown to the right with areas of necrosis indicated with arrows. Scale bar = 200 μm. (B). Immunofluorescent staining of livers from mice receiving either PBS or 1 × 10^6^ MSC for CD45 (green). Scale bar = 20 μm. Flow cytometric analysis of CCR1‐10 and CXCR1‐7 receptor expression (surface and total) shown as percentage of cell dissociation buffer (CDB)‐detached MSC positive for surface (open bars) and total (closed bars) expression (C left graph) with median fluorescence intensity (MFI) shown above bars (in bold for total expression). (C right graph) % of cells expressing chemokine receptors on their surface under basal conditions (white bar) or after TGFβ_1_ (hashed bar), IL4 (black bar) or IL10 (striped bar) stimulation for 24 h. MFI values are also shown (D left graph) and expressed as fold change over basal MFI levels of CCR in unstimulated MSC. Bars represent mean ± SEM of *n* = 5 donor samples. Quantitative analysis of total chemokine receptor gene expression levels in under basal conditions (white bar) or after TGFβ_1_ (hashed bar), IL4 (black bar) or IL10 (striped bar) stimulation were also measured by qPCR analysis (D right graph). Signal from stimulated MSC relative to endogenous β‐actin levels were expressed as fold change over basal levels of CCR in unstimulated MSC. Bars represent mean ± SEM of *n* = 3 different donor samples, performed in triplicate. *p < 0.05; **p < 0.01; ***p < 0.001; ****p < 0.0001. Statistical analysis was performed by Student's *t* test or ANOVA using Prism software. Data are expressed as mean with standard errors. A value of *p* < 0.05 was considered significant.

Infusion of 0.5 and 1 × 10^6^ MSC reduced serum ALT and tissue necrosis area 72 h after CCl_4_ administration as depicted in Figure 1A. This effect was associated with a concomitant reduction in inflammation with reduced numbers of hepatic CD45 positive cells (Figure 1B). To understand the potential role of MSC chemokine receptors in mediating engraftment to the injured liver their chemokine receptor expression profile was studied. A large percentage of MSC contained intracellular stores of CCR4 (95.84 ± 0.88%), CCR5 (67.96 ± 5.54%), and CXCR3 (92.69 ± 1.26%) with correspondingly high MFI values (Figure 1C,D). A smaller percentage of MSC expressed CCR6 (18.92 ± 7.56%), CCR9 (13.2 ± 7.16%), CCR10 (13.99 ± 6.39%), CXCR1 (22.1 ± 7.12%), and CXCR7 (25.02 ± 8.22%), albeit at lower levels. Supportive immune‐histochemical staining and basal gene expression for these receptors is presented in Figure S1.

### 3.2 Effects of cytokines on MSC chemokine receptor expression and engraftment in liver injury

**FIGURE 2:**
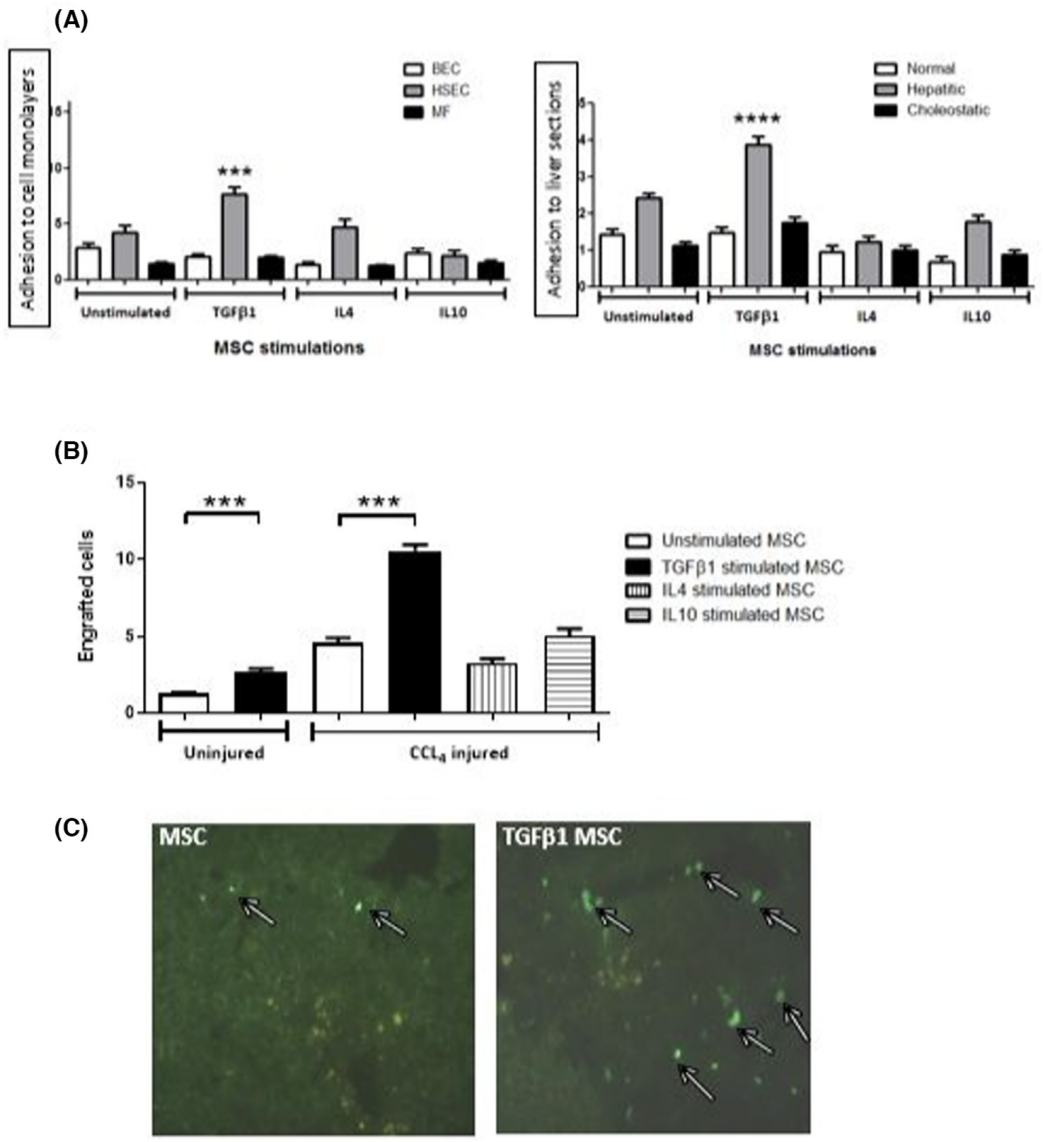
TGFβ_1_‐stimulated mesenchymal stromal cell (MSC) have superior hepatic recruitment after portal venous infusion. (A) Modified Stamper Woodruff assays showing basal adhesion of unstimulated MSC compared with TGFβ_1_, IL4 and IL10 stimulated MSC adhesion to human liver cells including, hepatic sinusoidal endothelial cells (HSEC, grey bars), biliary epithelial cells (BEC, white bars) and myofibroblast (MF, black bars) cell monolayers (left panel). Adhesion to liver sections from normal livers (white bars), hepatitic diseases (including Autoimmune hepatitis [AIH], Non‐alcoholic steatohepatitis,^29^ Alcohol related liver disease [ALD] grey bars) and cholestatic liver disease (including Primary sclerosing cholangitis [PSC] and Primary biliary cirrhosis [PBC], black bars). Bars represent area fraction covered by adherent CFSE‐labelled MSC in *n* = 3 samples using Image J analysis (left panel) or mean ± SEM cells/fov (right panel). (B) Stimulated (IL4, IL10 or TGFβ_1_) MSC engraftment in acutely CCl_4_ injured and uninjured C57Bl/6 mice relative to baseline unstimulated MSC engraftment, defined as 1. Data represent CFSE‐labelled MSC counted in 10 fields of view(fov) in four sections at four depths into the liver at ×40 magnification. Bars represent mean ± SEM of *n* = 3 donors and *n* = 6 mice. (C) Representative images of CCl_4_ injured C57Bl/6 mouse liver sections taken 15 min after infusion of control CFSE‐labelled MSC or TGFβ_1_‐stimulated MSC into liver via portal vein infusions. Scale bar = 20 μm. Representative of *n* = 6 mice at ×20 magnification. ***p < 0.001; ****p < 0.0001. Statistical analysis was performed by Student's *t* test or ANOVA using Prism software. Data are expressed as mean with standard errors. A value of *p* < 0.05 was considered significant.

Of a large panel of cytokines tested (Figure S2), only TGFβ_1_, IL4 and IL10 stimulation led to significant increases in the proportion of MSC expressing CCR4, CXCR3 and CCR5 (Figure 1C,D) by flow cytometry, although qPCR suggested no significant change in mRNA levels after stimulation (Figure 1D). Of these three cytokines only TGFβ_1_‐stimulated MSC demonstrated increased binding to cytokine‐stimulated (TNFα/IFNγ) human liver cell monolayers (HSEC, BEC and MF) or liver sections (Figure 2A). TGFβ_1_‐stimulated MSC (7.69 ± 0.59 cells per field of view [fov]; *p* < 0.001) exhibited increased adherence to stimulated HSEC compared with unstimulated MSC (4.18 ± 0.66 cells/fov), (Figure 2A, left panel). In addition, TGFβ_1_‐stimulated MSC were significantly more adherent to liver sections prepared from explanted diseased human livers of hepatitic nature, which was a pool of non‐alcoholic steatohepatitis/alcohol‐related liver disease cases (unstimulated 2.43 ± 0.13 cells/fov vs. stimulated 3.87 ± 0.23; *p* < 0.000) compared with cholestatic (primary biliary cholangitis/primary sclerosing cholangitis) sections (unstimulated 1.13 ± 0.11 vs. stimulated: 1.77 ± 0.13) and normal tissue (unstimulated 1.43 ± 0.15 vs. stimulated: 1.47 ± 0.16). Of note, IL4 and IL10 stimulation had no effect on MSC binding to liver sections (Figure 2A, right panel). To test adhesion and engraftment of MSC in injured liver in vivo CFSE‐labelled MSC were infused into control or acutely CCl_4_‐injured C57 Bl/6 mice via the portal vein. MSC were infused either unstimulated, or stimulated with TGFβ_1_, IL4 or IL10. We observed increased engraftment of TGFβ_1_‐stimulated MSC in injured mouse livers (2.29 ± 0.08 fold increase; *p* < 0.001) compared to unstimulated MSC (Figure 2B or C), whereas IL4 and IL10‐stimulation had no impact on engraftment.

### 3.3 TGFβ_1_ stimulation of MSC increases chemokine receptor expression, promotes redistributes redistribution chemokine receptors to the cell surface from the cytoplasm and enhances migration to their cognate ligands

**FIGURE 3:**
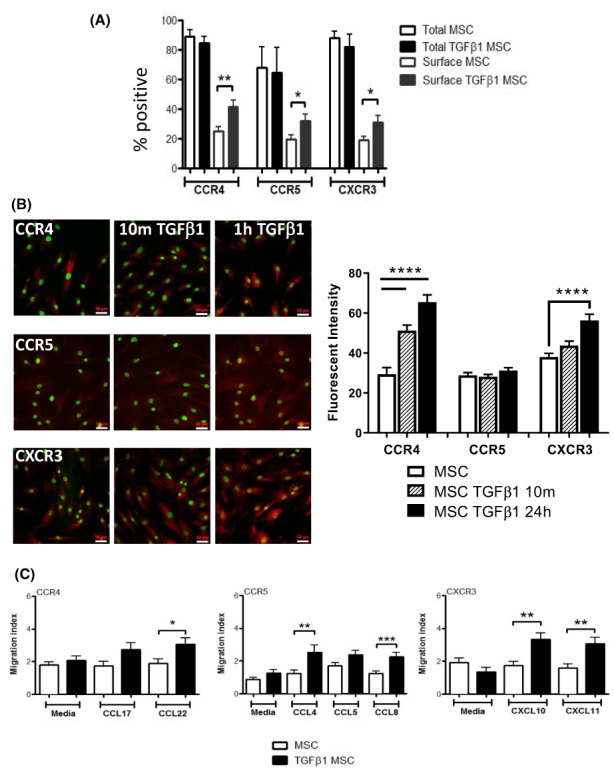
TGFβ_1_‐stimulated mesenchymal stromal cell (MSC) up‐regulate CXCR3 expression. (A) Flow cytometric analysis of total and surface CCR4, CCR5 and CXCR3 after TGFβ_1_ stimulation (black bars) compared with basal levels (open bars) for 24 h. Bars represent mean ± SEM of *n* = 5 donor samples. (B) Representative images of immunofluorescent staining of total CCR4, CCR5 and CXCR3 (red) with and without TGFβ_1_ (left column) stimulation are shown. MSC were grown on glass cover‐slips and nuclei were counter‐stained with DAPI (green, ×20 magnification). The fluorescent intensity of the cells was quantified from 5 different fields (20–40 cells/per field) at each time point, and the IF of each cell then plotted (right panel). (C) Migration of MSC and TGFβ_1_‐stimulated MSC to selected CCR4, CCR5 and CXCR3 chemokine ligands (as indicated) compared to media only controls was assessed using Boyden chambers. Data are expressed as Migration Index (cells/field of view). *p < 0.05; **p < 0.01; ***p < 0.001; ****p < 0.0001. Statistical analysis was performed by Student's *t* test or ANOVA using Prism software. Data are expressed as mean with standard errors. A value of *p* < 0.05 was considered significant.

**FIGURE 4:**
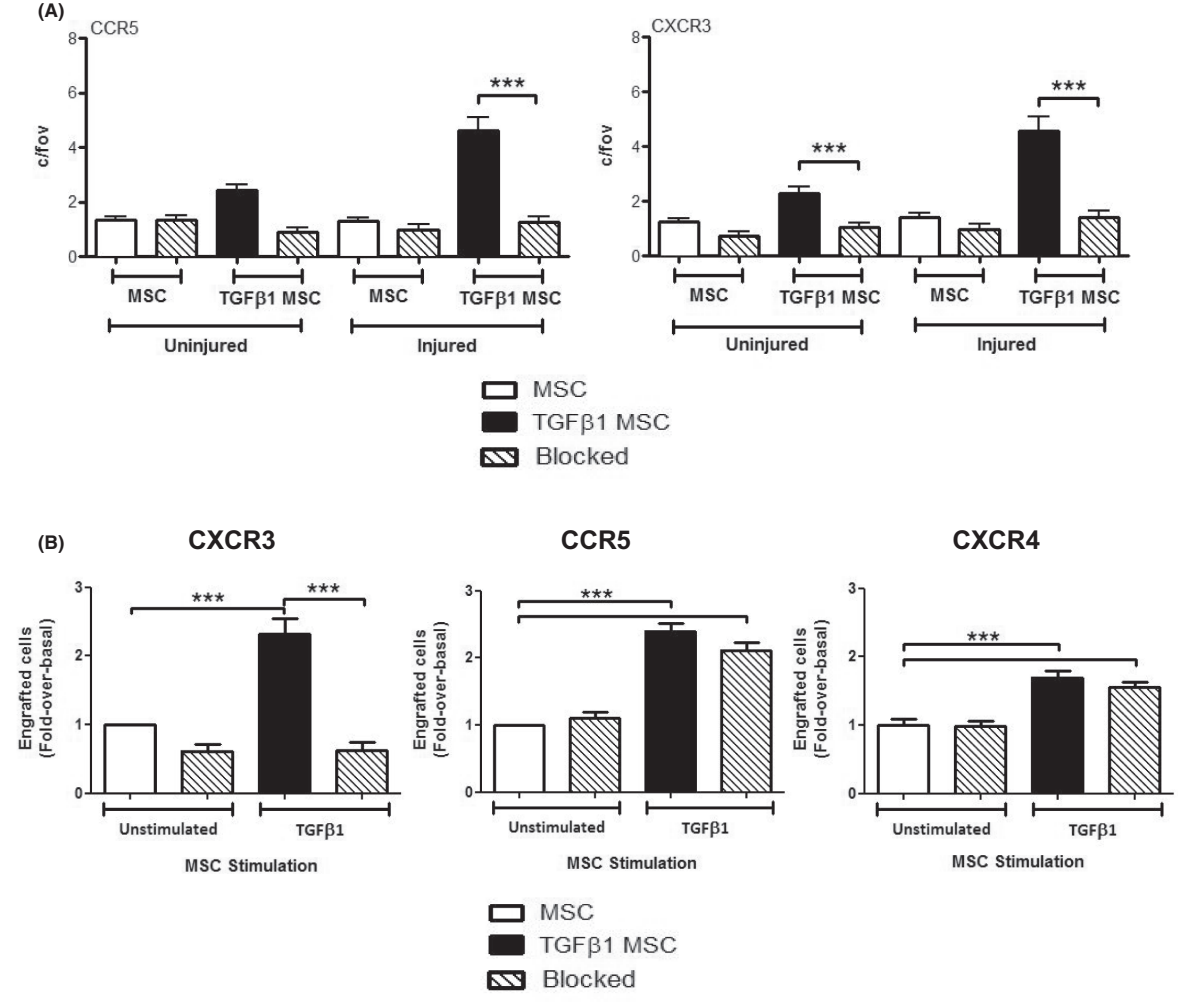
Enhanced hepatic engraftment of TGFβ_1_‐stimulated mesenchymal stromal cell (MSC) is mediated by functional up‐regulation of CXCR3. (A) Effect of CCR5 and CXCR3 blocking antibody on adhesion of TGFβ_1_‐stimulated MSC to uninjured or acute CCl_4_ injured mouse liver sections. Bars represent mean + SEM of adherent cells/fov for *n* = 3 donor samples. (B) The number of CFSE‐labelled MSC and TGFβ_1_‐stimulated MSC that engrafted in murine liver 72 h following portal vein injection was determined after treatment with function blocking antibodies to CXCR3 (10 μg/mL), CCR5 (10 μg/mL) or CXCR4 (10 μg/mL) or relevant IgG control. Data are represented relative to IgG control labelled unstimulated MSC, and bars represent mean ± SEM of *n* = 3 different donor samples. ***p < 0.001. Statistical analysis was performed by Student's *t* test or ANOVA using Prism software. Data are expressed as mean with standard errors. A value of *p* < 0.05 was considered significant.

Since TGFβ_1_‐stimulation of MSC (Figure 3A) increased surface expression of CCR4, CCR5 and CXCR3 without any change in mRNA levels, this suggested redistribution of these receptors to the cell surface. Confocal analysis confirmed redistribution of CXCR3 from the cytoplasm to the cell surface (Figure 3B right panel). Receptor redistribution was functional as TGFβ_1_‐stimulated MSC showed enhanced migration towards to CCL22 (3.07 ± 0.39 c/fov; *p* < 0.05) and the CCR5 ligands; CCL4 (unstimulated: 1.23 ± 0.21 c/fov vs. stimulated: 2.53 ± 0.45 c/fov; *p* < 0.01) and CCL8 (unstimulated: 1.23 ± 0.16 c/fov vs. stimulated: 2.27 ± 0.25 c/fov; *p* < 0.001), but not CCL5. The greatest increase in migration after TGFβ_1_ stimulation was in response to the CXCR3 ligands CXCL10 (unstimulated: 1.73 ± 0.26 c/fov vs. stimulated: 3.33 ± 0.41 c/fov; *p* < 0.01) and CXCL11 (unstimulated: 1.60 ± 0.25 c/fov vs. stimulated: 3.07 ± 0.40 c/fov; *p* < 0.01) (Figure 3C). As migration of MSC towards CXCR3 ligands and CCR5 was most impressive after TGFβ_1_ stimulation, we used function blocking antibodies for these receptors in Stamper Woodruff assays (Figure 4). TGFβ_1_‐stimulated MSC bound in significantly higher numbers (4.60 ± 0.50 c/fov) to injured mouse liver sections compared to unstimulated MSC (1.29 ± 0.13 c/fov), and CCR5 and CXCR3 blockade reduced binding to injured liver sections back to basal levels (Figure 4A). We then infused CFSE‐labelled MSC into CCl_4_‐injured mice via the portal vein, and observed increased engraftment of TGFβ_1_‐stimulated MSC in mouse livers. Whilst blocking CXCR3 on unstimulated MSC had no effect on their engraftment in injured mouse livers, there was a marked effect on TGFβ_1_‐stimulated MSC with engraftment reducing from a 2.32 ± 0.22 fold increase from baseline to a 0.63 ± 0.11 fold reduction (*p* < 0.001; Figure 4B). In contrast, blockade of CXCR4 and CCR5 blockade on MSC had no effect on engraftment of either control or stimulated MSC in injured mouse livers. To define the duration of TGFβ_1_ exposure required to induce CXCR3 expression we looked after 10 min, 1, 4 and 24 h stimulation. At 24 h there was a marked increase in surface CXCR3 expression by flow cytometric and confocal analysis. It is important to highlight however, that transcriptional upregulation of chemokine expression cannot be completely excluded although the changes seen within 24 h suggest the receptor mobilization plays a much more significant role following cytokine treatment.

### 3.4 Systemically administered TGFβ_1_ ‐stimulated MSC home specifically to the injured liver

**FIGURE 5:**
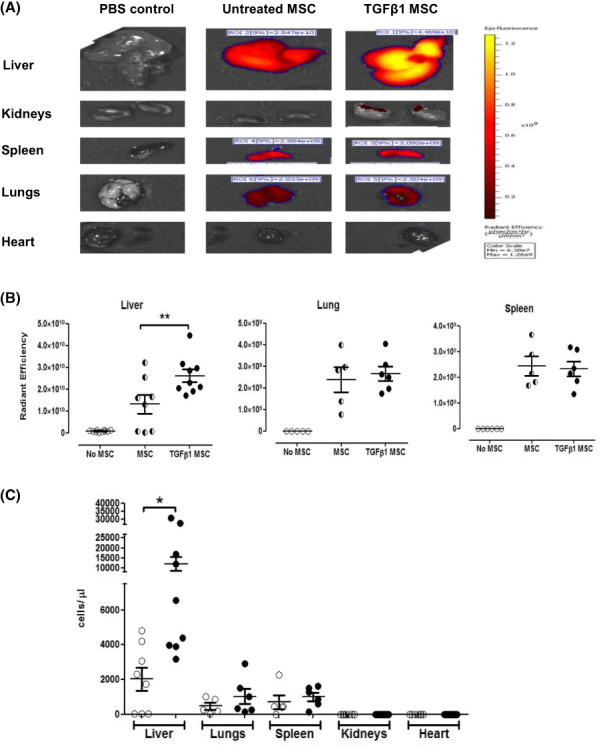
Enhanced engraftment of TGFβ_1_‐stimulated mesenchymal stromal cell (MSC) is specific to liver injury. (A) Representative IVIS images of acute CCl_4_ injured excised mouse organs 72 h after infusion of PBS (left), Dir‐red labelled MSC (middle) or Dir‐red labelled TGFβ_1_‐stimulated MSC^14^ via tail‐vein. This is represented quantitatively in (B), with open circles for PBS infusion, mixed circles for unstimulated MSC and black circles for TGFβ_1_‐stimulated MSC. (C) Flow cytometric analysis of digested CCl_4_‐injured C57Bl/6 organs for absolute numbers of Dir‐red labelled unstimulated MSC (open circles) or TGFβ_1_‐stimulated (black circles) MSC, left panel. Lines represent mean ± SEM of *n* = 9 different donor samples. Weights of organs are depicted in right panel for the different groups. *p < 0.05; **p < 0.01. Statistical analysis was performed by Student's *t* test or ANOVA using Prism software. Data are expressed as mean with standard errors. A value of *p* < 0.05 was considered significant.

Mice were acutely injured with CCl_4_ and 4 h later received Direct red‐labelled untreated MSC or TGFβ_1_ stimulated MSC (or PBS control). After 68 h, murine liver fluorescence (Radiant Efficiency) was quantified using an IVIS imager and there were significantly higher levels of fluorescence in the livers of mice receiving TGFβ_1_‐stimulated MSC (Figure 5A). Fluorescent activity was also detected in the lungs (2.38 × 10^9^ ± 5.81 × 10^8^), liver (1.31 × 10^10^ ± 4.29 × 10^9^) and spleen (2.44 × 10^9^ ± 3.72 × 10^8^) with minimal activity in the kidneys and heart. Notably TGFβ_1_ stimulation of MSC specifically increased their liver homing (2.61 × 10^10^ ± 2.87 × 10^9^; *p* < 0.01) with no increase in levels of fluorescence activity elsewhere (Figure 5B). These findings were also validated by FACS analysis of single cell digests of harvested organs. Again significantly greater numbers of MSC were retrieved from the livers from TGFβ_1_‐stimulated MSC‐treated mice (12,146 ± 3569 cells/μL) compared to untreated MSC (2024 ± 676.4 cells/μL; *p* < 0.05). As with the IVIS analysis there was no significant difference between numbers of MSC or TGFβ_1_‐stimulated MSC in lungs or spleen (Figure 5C).

### 3.5 TGFβ_1_ ‐stimulated MSC reduce liver inflammation, necrosis and liver serum aminotransferase levels in a mouse model of liver damage

**FIGURE 6:**
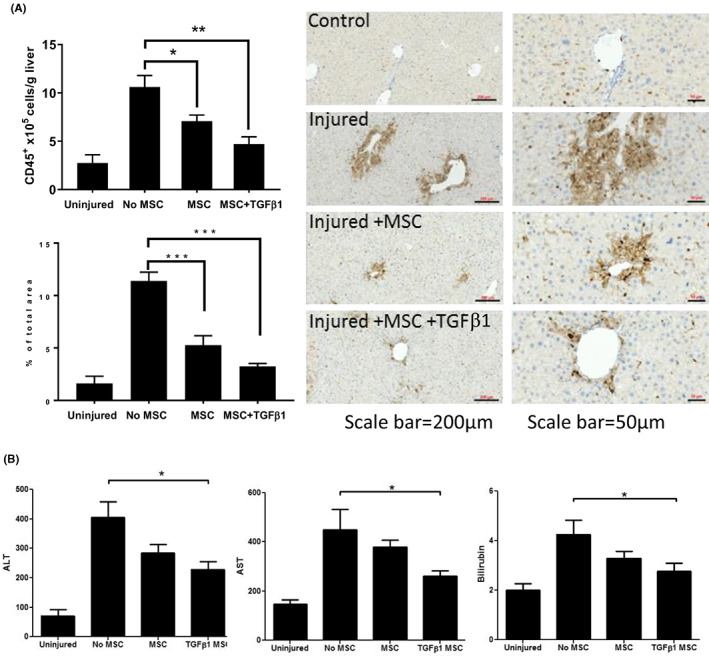
TGFβ_1_ stimulation of mesenchymal stromal cell (MSC) enhances their ability to reduce liver injury after CCl_4_ injury. (A) Immunohistochemical analysis of CD45^+^ cells^59^ in acute CCl_4_ injured C57Bl/6 mouse livers 72 h after PBS (untreated), MSC or TGFβ_1_‐stimulated MSC infusion. Data represent CD45^+^ cells counted in 10 fields of view at ×40 magnification.^14^ Bars represent mean ± SEM of *n* = 3 donors. (B) Serum levels of ALT, AST and Bilirubin in same studies. Groups represent mean ± SEM of *n* = 9 mice. *p < 0.05; **p < 0.01; ***p < 0.001. Statistical analysis was performed by Student's *t* test or ANOVA using Prism software. Data are expressed as mean with standard errors. A value of *p* < 0.05 was considered significant.

To determine the impact of MSC infusion on the pathogenesis of CCl_4_‐induced injury, livers were harvested 72 h after infusion. Mice receiving untreated MSC had fewer CD45^+^ cells (33.18 ± 1.68 c/fov; *p* < 0.05) than control mice (42.27 ± 3.06 c/fov), whilst those receiving TGFβ_1_‐stimulated MSC had the fewest CD45^+^ cells (20.02 ± 1.80 c/fov; *p* < 0.001, Figure 6A). Similarly, the injury‐associated increase in serum ALT levels was less pronounced in mice receiving TGFβ_1_‐stimulated MSC (228.1 ± 26.52 IU/L; *p* < 0.05) as compared to PBS‐treated mice (404.7 ± 53.62 IU/L, Figure 6B). Serum Bilirubin levels were also reduced in mice receiving TGFβ_1_‐stimulated MSC (2.77 ± 0.33 IU/L; *p* < 0.05) compared to PBS‐treated mice (4.25 ± 0.58 IU/L). Similarly AST levels were also reduced with TGFβ_1_‐stimulated MSC (265.5 ± 20.66 IU/L; *p* < 0.05) as compared to PBS‐treated mice (394.5 ± 45.49 IU/L) as shown in (Figure 6B).

### 3.6 Infusion of unstimulated and TGFβ1‐stimulated MSC after acute CCl_4_ injury results in a reduction in M1‐like/M2‐like ratio of hepatic macrophages

**FIGURE 7:**
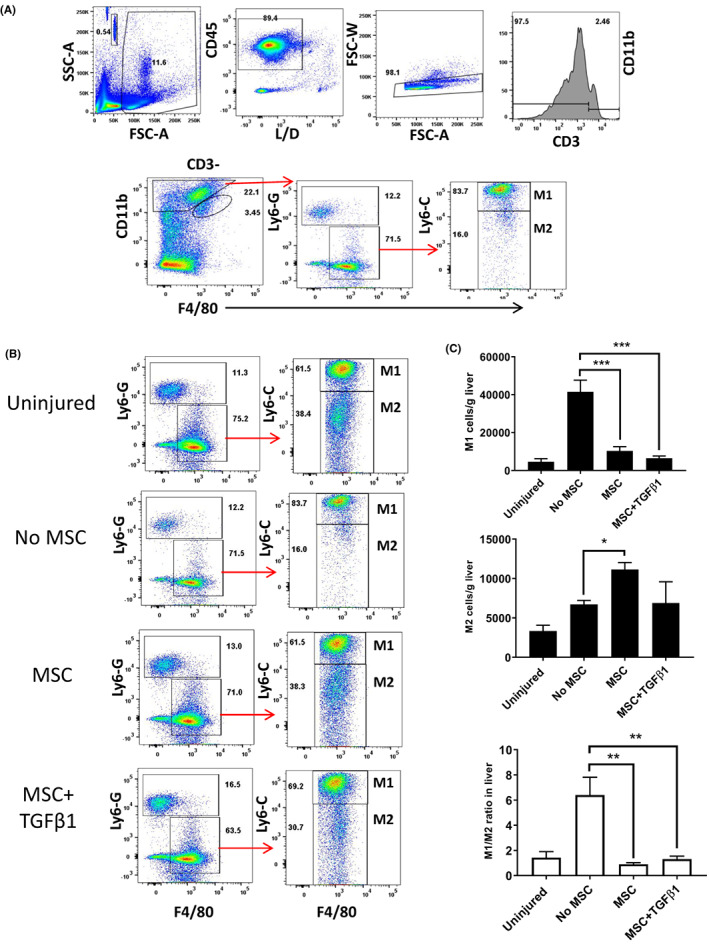
Mesenchymal stromal cell (MSC) infusions result in a decrease in M1/M2‐like macrophage ratio. (A) Flow cytometric gating of digested murine livers to determine macrophage populations using the following strategy: M1‐like macrophages (gated CD45^+^CD3^−^CD11b^+^F4/80^+^Ly‐6G‐Ly6C high) and M2‐like macrophages (gated CD45^+^CD3^−^CD11b^+^F4/80^+^Ly‐6G‐Ly6‐C low). (B or C) Representative flow cytometry plots are shown from livers digested 72 h following acute CCl_4_ injury for M1 and M2‐like macrophages. Values are expressed as number/gram of liver tissue. The ratio of M1 and M2‐like macrophages was calculated (right panel). *p < 0.05; **p < 0.01; ***p < 0.001. Statistical analysis was performed by Student's *t* test or ANOVA using Prism software. Data are expressed as mean with standard errors. A value of *p* < 0.05 was considered significant.

The impact of infusions of MSC on macrophage numbers and polarization was assessed by flow cytometric quantification of digested murine livers (Figure 7A). Both unstimulated and TGFβ1‐stimulated MSC resulted in reductions of the numbers of M1‐macrophages (gated CD45^+^CD3^−^CD11b^+^F4/80^+^Ly‐6G‐Ly‐6C high) with a variable increase in M2‐macrophages (gated CD45^+^CD3^−^CD11b^+^F4/80^+^Ly‐6G‐Ly6‐C low) as well as an overall reduction in the Ly‐6C^hi^/Ly‐6C^lo^ (M1‐like to M2‐like) ratio within the liver (Figure 7B or C).

### 3.7 TGFβ_1_ stimulation of MSC enhances their ability to suppress T cell proliferation in a PGE2‐dependent fashion

**FIGURE 8:**
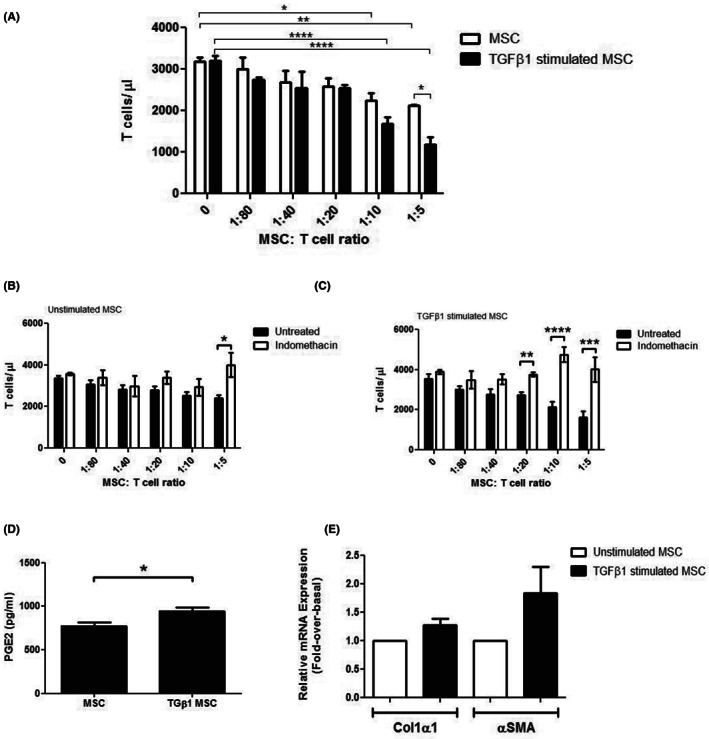
TGFβ_1_ stimulation of mesenchymal stromal cell (MSC) enhances their ability to suppress T cell proliferation in a PGE_2_‐dependent fashion. (A) Ability of TGFβ_1_‐stimulated MSC to inhibit proliferation of co‐cultured activated CD3^+^CD4^+^CD25^−^ T effector cell was determined flow cytometrically. At a ratio of 1:5 MSC to T effector cells, stimulation with TGFβ_1_ conferred greater efficacy to MSC. (B or C) Ability of both unstimulated and TGFβ_1_‐stimulated MSC was abrogated by addition of indomethacin (50 μM). (D) Mesenchymal stromal cell were stimulated with TGFβ1 for 24 h before supernatants were collected and PGE2 levels measured using a sandwich ELISA. Bars represent mean ± SEM of *n* = 3 different donors. (E) Analysis of total collagen‐1 and αSMA gene transcription in TGFβ_1_‐stimulated MSC, measured by Real Time Polymerase Chain Reaction (qPCR) analysis, and expressed as fold change over basal levels of in unstimulated MSC (open bars). Bars represent mean ± SEM of *n* = 3 different donor samples, performed in triplicate. There was no significant effect of 24 h stimulation with TGFβ_1_ on either Col1 or αSMA gene expression in MSC. *p < 0.05; **p < 0.01; ***p < 0.001; ****p < 0.0001. Statistical analysis was performed by Student's *t* test or ANOVA using Prism software. Data are expressed as mean with standard errors. A value of *p* < 0.05 was considered significant.

TGFβ_1_‐stimulated MSC also demonstrated a greater ability to inhibit proliferation of co‐cultured, activated CD3^+^CD4^+^CD25^−^ T effector cells in vitro (Figure 8A) which were abrogated by the addition of the non‐steroidal anti‐inflammatory drug indomethacin (Figure 8B or C). Indomethacin acts as a nonselective cyclooxygenase (COX) inhibitor that interferes with prostaglandin E_2_ biosynthesis thereby interfering with leucocyte proliferation/activation. Moreover, MSC stimulated with TGFβ_1_ for 24 h secreted greater amounts of PGE_2_ (Figure 8D) than unstimulated MSC. Quantitative analysis of total collagen‐1 and αSMA gene levels in stimulated MSC demonstrated no significant effect of TGFβ_1_ on either Col1 or αSMA expression.

## 4 DISCUSSION

We have demonstrated that TGFβ_1_ stimulation of MSC more than doubles their homing to the acutely injured liver and is associated with a resultant further reduction in inflammation and hepatic damage. Increased hepatic homing is mediated by a TGFβ_1_‐dependent increase in MSC surface expression of CXCR3, which promotes binding to hepatic endothelium in vitro and organ‐specific migration to the injured liver in vivo. Use of TGFβ_1_ stimulation to enhance MSC function represents a novel strategy to improve therapeutic use of MSC in inflammatory liver injury.

Previous studies have reported modest beneficial effects of rodent and human MSC in models of liver injury such as carbon tetrachloride^3^, ^32^, ^33^ galactosamine,^5^, ^6^ chemical‐induced primary biliary cirrhosis^4^ or models of hepatic transplantation.^34^, ^35^ Our data provide additional support for the efficacy of unprimed human MSC in liver injury, but demonstrate that significantly greater efficacy can be achieved by cytokine priming. Use of rodent MSC in such models causes improvements in liver damage which appear to be, in part mediated by a reduction in oxidative stress^36^ and cellular infiltrates.^6^ Human MSC infusions have been reported to show similar benefit in CCl_4_ injury^3^, ^32^ although the mechanism of action is unclear apart from reduction in oxidative stress. Many have reported that MSC may exert their anti‐inflammatory actions remotely through either the release of mediators such as TSG6^37^ and/or modulation of circulating effectors such as myeloid derived suppressor cells.^38^ These findings do not preclude an added action of MSC at the site of injury, and in that regard enhanced hepatic homing is a logical target.

We have previously shown that trypsin‐detached MSC use β_1_‐integrin and CD44 to mediate hepatic engraftment^9^ and others have reported they use chemokine receptor CXCR4 for migration/engraftment in other settings.^39^, ^40^ Notably, we have demonstrated that the method of cell detachment is critical in preserving basal chemokine receptor expression on MSC.^18^ Our data demonstrate that whilst priming with IL4/IL10/TGFβ_1_ can significantly increase surface expression of a range of chemokine receptors, only TGFβ_1_‐stimulated MSC displayed an increased hepatic recruitment in both in vitro and in vivo settings (Figure 2B), and this effect appeared to be mediated by the increased surface expression of CXCR3. Notably increased organ homing following TGFβ_1_‐stimulation was liver‐specific, in keeping with other studies,^41^, ^42^ reflecting the targeting of infused cells to the inflamed site. There is precedent for such a role for CXCR3 as Curbishley et al.,^43^ have previously demonstrated that CXCR3 expression is the major determinant for lymphocyte adhesion/trans‐migration in the injured liver. Thus, similar mechanism may operate to maintain surface expression of CXCR3 on MSC through inhibition of degradation and internalization.^44^ Other mechanisms implicated in the TGFβ induced increased expression of CCRs include activation of p38/MAPK signalling pathways, as seen in immune cells^45^ and inhibition of Metalloproteinases, involved in cleavage of CCRs at the cell surface.^46^ Our data suggest that TGFb stimulation does not have a major impact on transcriptional regulation (Figure S2B) indicating that in this setting the dominant mechanism driving increased chemokine expression on our MSCs is recirculation or inhibition of MMP cleavage.

Recent studies suggest that allogeneic MSC, although hypo‐immunogenic, are not intrinsically immune privileged and that allogeneic MSC induce a memory T‐cell response resulting in rejection.^47^ Although human MSC are even more likely to generate an immune response after infusion into mice, we did not see an increase in CD45^+^ cells within the liver in our acute injury models and thus this approach provides an important method for obtaining in vivo data relevant for subsequent clinical trials, especially given the knowledge that human MSC use different mechanisms to immunomodulate compared to murine MSC.^48^

In our study, stimulation with TGFβ_1_ had no discernible effect on other properties of MSC including differentiation to myofibroblasts, and importantly MSC were cleared rapidly after infusion, rendering it highly unlikely that they could contribute directly to fibrogenesis. This also suggests that repeated infusions of pre‐stimulated cells may prolong benefit without increasing risk of fibrosis. A recent study^49^ and comprehensive review^50^ indicate that adoptively transferred MSC make no contribution to fibrosis, despite contrasting studies,^51^, ^52^ which is in keeping with our data. Indeed, adoptively transferred MSC have been shown to induce a reduction in fibrosis^53^ when infused in models of chronic liver damage with CCl_4_. This effect would appear to be mediated by blockade of Dlk1 activation thus causing a reduction in activation of hepatic stellate cells,^53^ along with increased MMP13 activity promoting fibrinolysis within the liver.^54^

A range of mechanisms have been reported by which MSC can mediate their immunomodulatory effects: MSC inhibit T cell activation induced by an anti‐CD3/CD28 antibody stimulus, mitogens, and allo‐antigens. They also inhibit NK cell activation, as well as B cell terminal differentiation, and dendritic cell maturation and functionality. In addition, MSC can inhibit homing of immune cells to lymph nodes and impair T‐cell priming in vivo.^8^, ^55^ However the precise molecular mechanisms responsible for the anti‐inflammatory effects of MSC in liver disease are still unknown, although MSC can reduce oxidative stress^3^ and CD45 infiltration.^6^ We saw a reduced CD45^+^ infiltrate after administration of MSC, by immunohistochemistry and flow cytometric analysis of digested murine liver, which correlated with reduced tissue necrosis and ALT in serum. Our data suggest that TGFβ_1_ stimulation also enhances the ability of MSC to suppress T cell proliferation or recruitment, and thus this may be a factor in the superior efficacy seen with primed cells. However, further work is required to establish whether the efficacy seen with TGFβ_1_‐dependent priming of MSC is predominantly driven by enhanced immunomodulatory action of MSC or their increased hepatic homing.

Furthermore, our data indicate that hepatic macrophage profile changes significantly following administration of MSC, with or without, TGFβ_1_ stimulation. Our data indicate that MSC infusion is associated with a reduction in differences in the proportion of macrophage subsets expressed as a ratio of Ly‐6C^hi^/Ly‐6C^lo^ (M1‐like to M2‐like) macrophages. The differential expression of Ly‐6C has been used to identify monocyte subsets in rodent models of liver injury where Ly‐6C^hi^ monocytes exhibit pro inflammatory phenotype (M1) and Ly‐6C^−^ monocytes exhibit the restorative phenotype (M2).^56^ As recognized by the literature, surface marker expression of macrophages is likely to be more complex and dynamic and thus even more extensive panels (CD163, CD206, CD68 and TLR4) do not completely characterize the full phenotype of macrophages in vivo^57^ and our panel is acceptable with these caveats.^58^ MSC have also been reported to mediate some of their anti‐inflammatory effects by inducing secretion of IL10 from macrophages^59^ and by inducing an M2 phenotype in unpolarised monocytes.^60^ Indeed phagocytosis of MSC by monocytes can trigger acquisition of an immunosuppressive M2 phenotype which enhances the immunoregulatory response to MSC infusion.^61^ Thus, some of the hepatic M2 macrophages (Figure 7C) present after MSC treatment may have differentiated locally in response to phagocytosis of hepatic MSC. However, there is also evidence that lung‐resident monocytes can also phagocytose trapped MSC and differentiate to regulatory macrophages which can then migrate to distant sites.^61^ Given we did indeed see a background level of MSC entrapment in the lungs (Figure 5), it is also possible that cells trafficking from this site could contribute to the pool of M2 macrophages we identified in our injured livers. Thus, in our model, the hepato‐protective effects of TGFβ_1_ primed MSC may be linked to a direct suppression of T cell activation and recruitment, and enhanced macrophage recruitment and differentiation within the liver, thus shifting the hepatic microenvironment towards a more reparative situation. Further study of the phenotype of hepatic myeloid cell subsets would be of value. In conclusion, we have demonstrated that priming of MSC with TGFβ_1_‐ enhances hepatic homing and anti‐inflammatory efficacy, without evidence of off‐target effects. This provides new opportunities to develop more clinically effective regimens of MSC therapy in clinical trials.

Potential limitations include the heterogeneity of BM MSCs used due to batch to batch variation from different donors, stem cell aging and associated vulnerability,^62^ which can affect the conclusions drawn. To address this we used BM MSC from a minimum of three independent donors in our studies. Also BM MSC from Lonza are in themselves pooled samples from multiple donors which minimizes some of the afore‐mentioned risks. There is evidence that alternative sources of MSCs such as human‐induced pluripotent stem cells (hiPSCs) may be less impacted by aging with higher potency in immunomodulatory properties.^63^

## AUTHOR CONTRIBUTIONS

**Abhilok Garg:** Data curation (lead); formal analysis (lead); investigation (lead); methodology (lead); writing – original draft (lead). **Sheeba Khan:** Writing – original draft (supporting); writing – review and editing (supporting). **N. Luu:** Formal analysis (supporting); investigation (supporting); supervision (supporting); validation (supporting). **Davies J. Nicholas:** Data curation (supporting); formal analysis (supporting); investigation (supporting); supervision (supporting); visualization (supporting); writing – review and editing (supporting). **Victoria Day:** Funding acquisition (supporting); resources (supporting). **Andrew L. King:** Writing – review and editing (supporting). **Janine Fear:** Data curation (supporting); formal analysis (supporting); investigation (supporting); validation (supporting). **Patricia F. Lalor:** Conceptualization (lead); data curation (supporting); formal analysis (supporting); funding acquisition (lead); investigation (supporting); methodology (supporting); project administration (lead); resources (lead); software (supporting); supervision (lead); validation (supporting); visualization (lead); writing – original draft (supporting); writing – review and editing (supporting). **Philip N. Newsome:** Conceptualization (lead); data curation (supporting); formal analysis (supporting); funding acquisition (lead); investigation (supporting); methodology (supporting); project administration (supporting); resources (lead); software (supporting); supervision (lead); validation (supporting); visualization (supporting); writing – original draft (supporting); writing – review and editing (supporting).

## FUNDING INFORMATION

This work was supported by University Hospital Birmingham Charities. PNN is supported by the NIHR Birmingham Biomedical Research Centre based at University Hospitals Birmingham and the University of Birmingham. The views expressed are those of the author and not necessarily those of the NHS, the NIHR or the Department of Health.

## CONFLICT OF INTEREST STATEMENT

There are no relevant disclosures.

## Supporting information


Figures S1–S4

Appendix S1.

## Data Availability

The data that support the findings of this study are available from the corresponding author upon reasonable request.
