# Supplementary material for: TGFβ1 priming enhances CXCR3‐mediated mesenchymal stromal cell engraftment to the liver and enhances anti‐inflammatory efficacy
Source: J Cell Mol Med. 2023 Feb 23;27(6):864–78. doi: 10.1111/jcmm.17698 (PMC10002976; doi:10.1111/jcmm.17698)
Supplement: Supplementary file 1 — Figures S1–S4 [file JCMM-27-864-s002.zip › JCMM_17698_Supp Figures 1-4_SK edit 30_jan.docx]

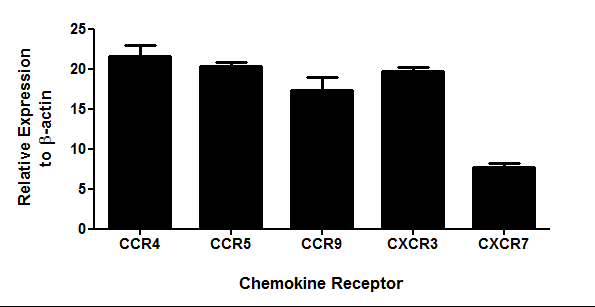


Supplementary figure 1

A

B


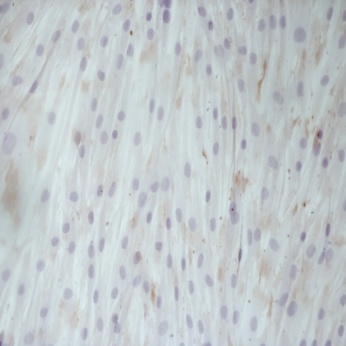


CCR9


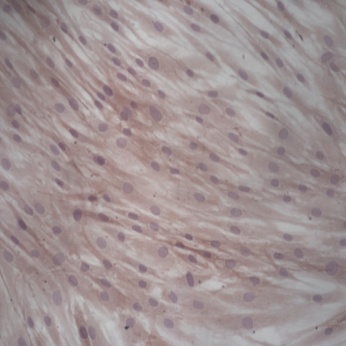


CXCR3


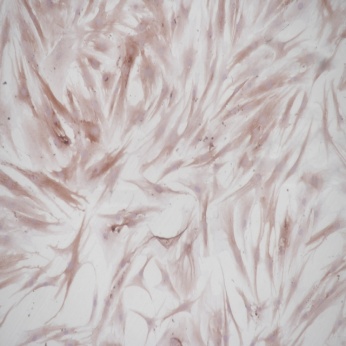


CCR4


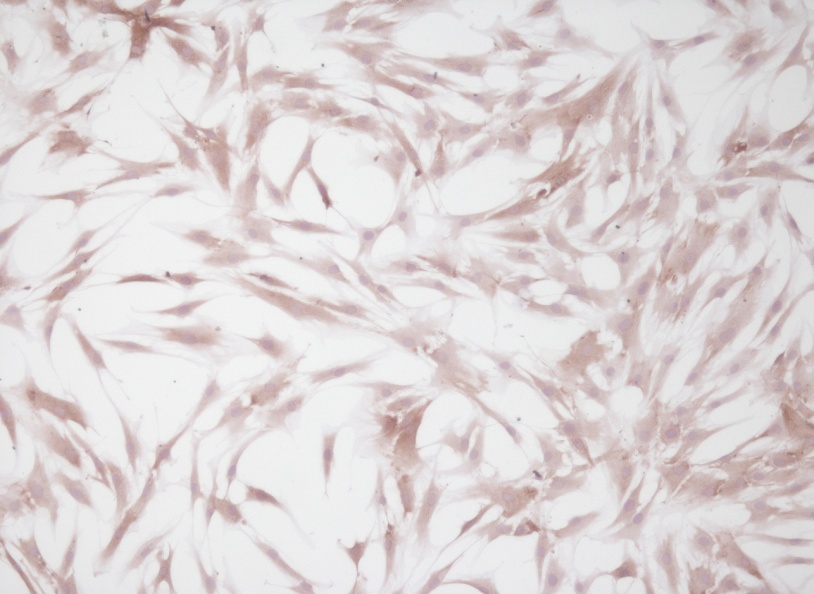


CCR5


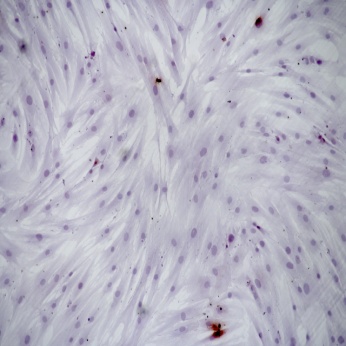


CXCR7


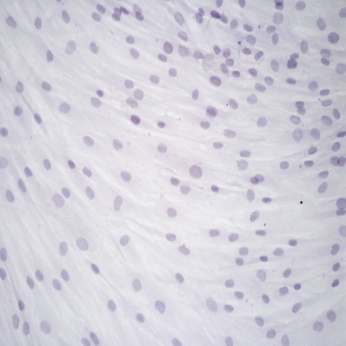


Control

(i)

(ii)

(iii)

(iv)

(v)

(vi)

100μm

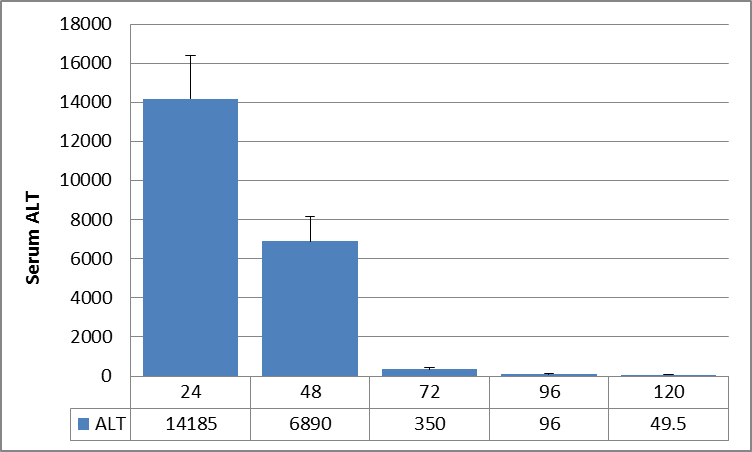

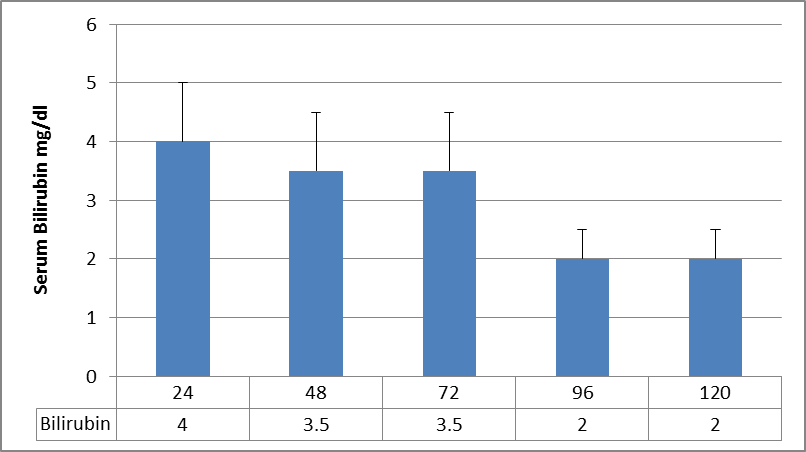


24h

48h

72h

96h

120h

Supplementary figure 3

A

B

C


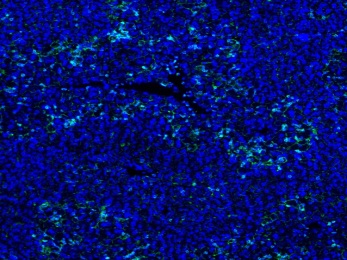

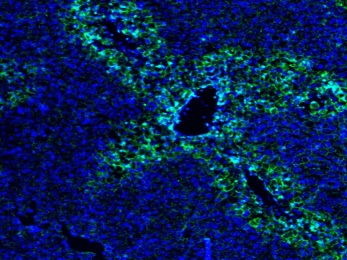

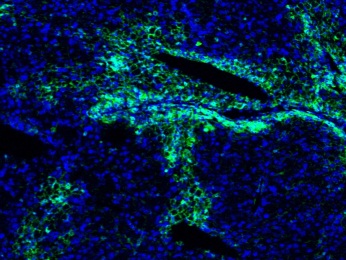

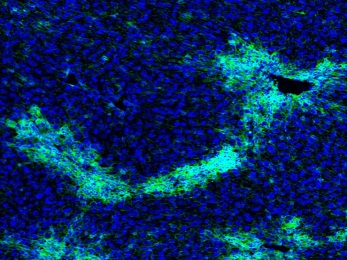

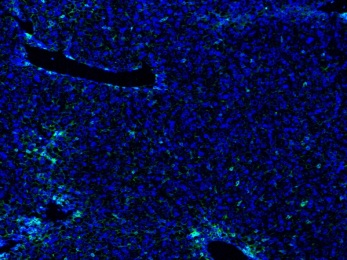

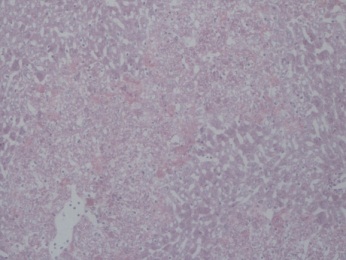

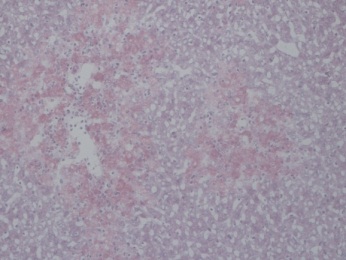

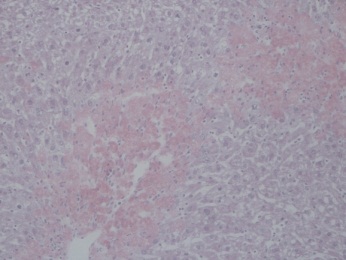

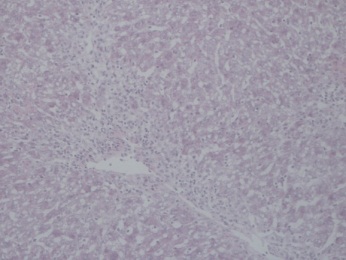

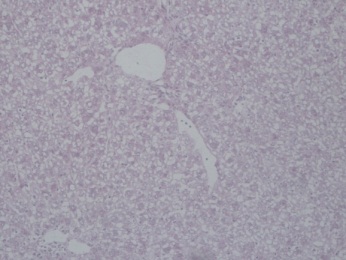

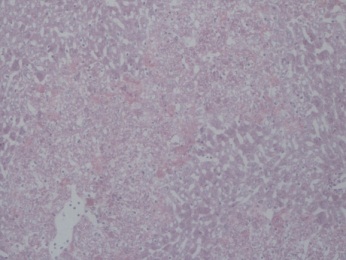


200μm


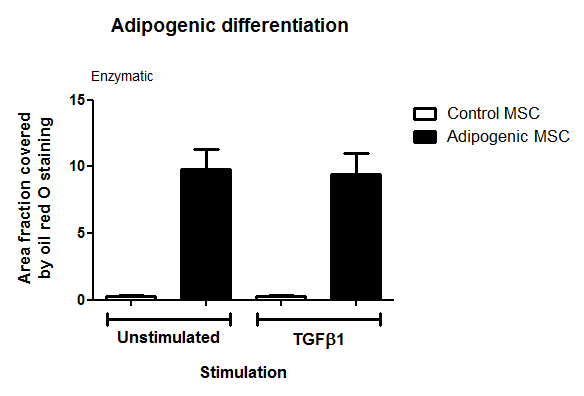

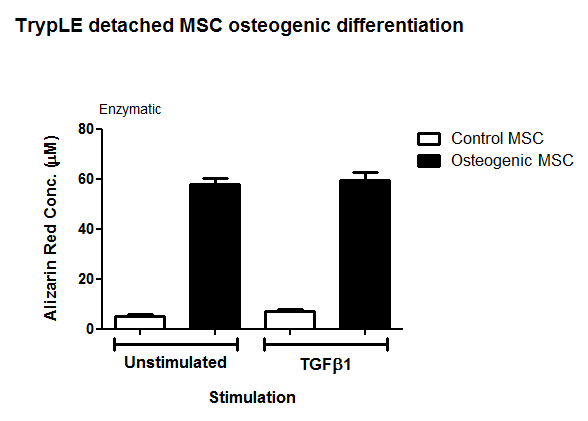


Supplementary figure 4


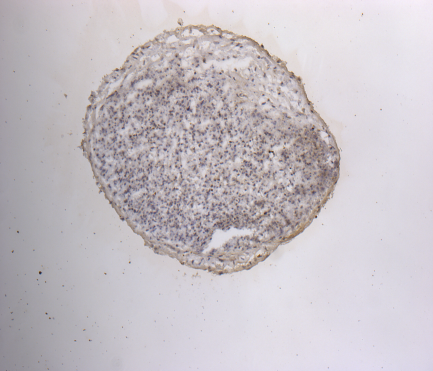

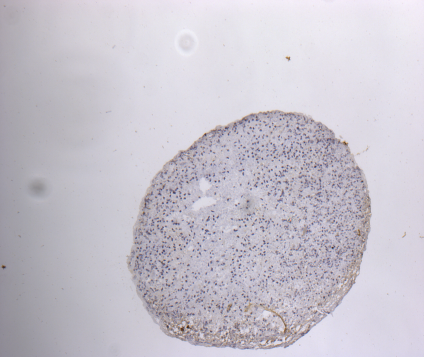

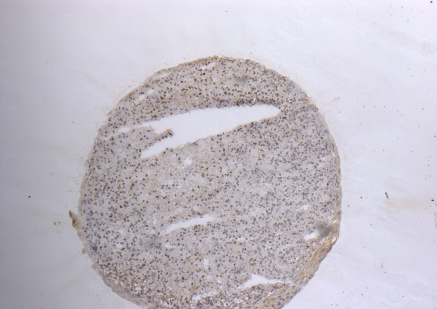


**MSC**

**TGFβ1**

**Control**

**Test**

**IgG**

**IgG**

**Col II**


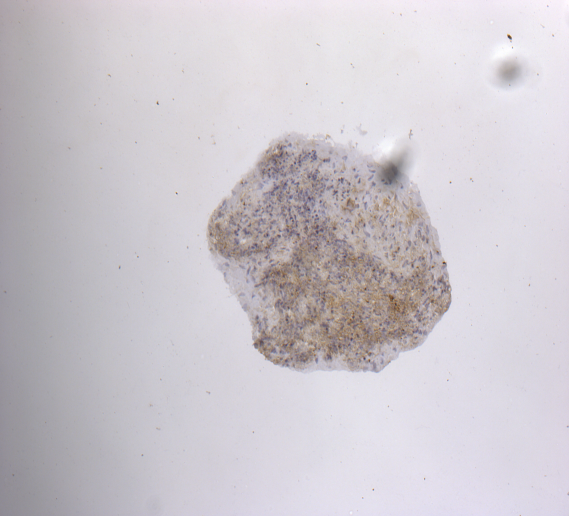

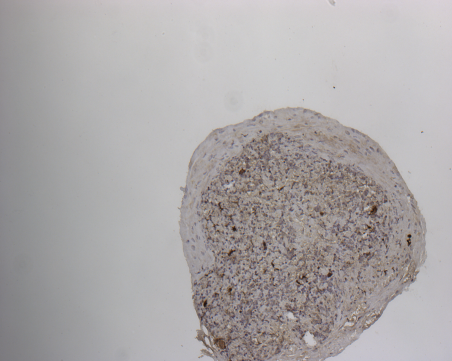

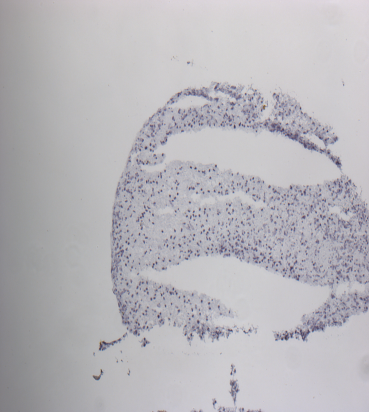

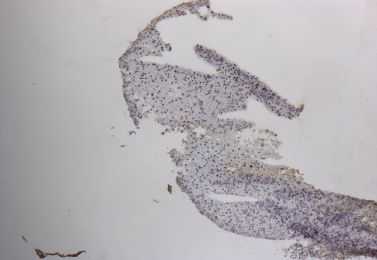

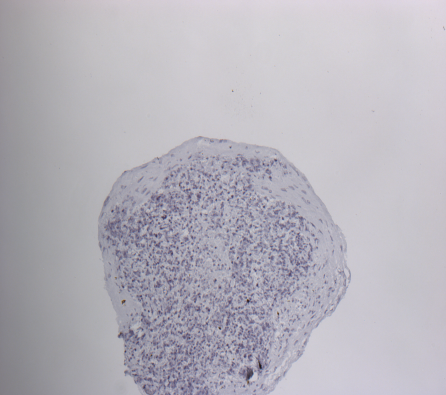


**Col II**

A

B

C
