# Supplementary material for: TGFβ1 priming enhances CXCR3‐mediated mesenchymal stromal cell engraftment to the liver and enhances anti‐inflammatory efficacy
Source: J Cell Mol Med. 2023 Feb 23;27(6):864–78. doi: 10.1111/jcmm.17698 (PMC10002976; doi:10.1111/jcmm.17698)
Supplement: Supplementary file 1 — Figures S1–S4 [file JCMM-27-864-s002.zip › JCMM_17698_Supplementary figure_legends_SK edit.docx]

**Supplementary Figure 1**

**A:** Representative images of immunohistochemical staining with Nova red (red/ brown) of CCR9, CXCR3, CCR4, CCR5 and CXCR7 compared to concentration matched controls. MSC are grown on glass cover-slips and counterstained with Mayer’s Haematoxylin (n=3 donors at x20 magnification). **B:** Quantitative analysis of total CCR4, CCR5, CCR9, CXCR3 and CXCR7 gene levels in MSC, measured by Real Time Polymerase Chain Reaction (qPCR) analysis and expressed as relative to endogenous MSC β-actin levels. The results were calculated by the comparative threshold cycle (Ct) method, with the Ct for β-actin used to normalise the results. Expression of each gene was calculated with the endogenous level of β-actin in MSC defined as 1. Bars represent mean + SEM of n=3 different donor samples, performed in triplicate.

**Supplementary Figure 2**

**A:** Flow cytometry analysis of CCR4, CCR5, CXCR3, CCR9, CXCR4 and CXCR7 receptor expression after stimulation with a selection of cytokines shown as percentage of CDB detached MSC positive for surface (open bars) and total (closed bars) expression with median fluorescence intensity (MFI) shown for selected CCR. Striped column represents intracellular expression of CCR in unstimulated MSC. Bars and MFI represent mean + SEM of n=3 different donor samples. **B:** Quantitative analysis of total CCR4, CCR5, CXCR3, CCR9, CXCR4 and CXCR7 gene expression levels in MSC after cytokine stimulation as measured by qPCR analysis. Stimulated MSC gene levels relative to endogenous β-actin levels in stimulated MSC were expressed as fold change over basal levels of CCR in unstimulated MSC. Bars represent mean + SEM of n=3 different donor samples, performed in triplicate.

**Supplementary Figure 3**

Following administration of a single dose of CCl_4_ a time course looking at liver histology (H&E and CD45 immunofluorescent staining (**A**)), serum ALT (**B**) and serum bilirubin (**C**) was undertaken.

**Supplementary Figure 4**

**A:** Image J analysis of oil red O staining of enzyme and non-enzyme detached TGFβ_1_ stimulated MSC differentiated into adipocytes. Bars represent oil red O stained mean area fraction + SEM of n=3 samples. **B:** Alizarin red staining of enzyme and non-enzyme detached TGFβ_1_ stimulated MSC differentiated into osteoblasts. Bars represent mean calcium concentration (µM) + SEM of n=3 samples. **C:** Representative images, DAB staining of collagen II as a marker of chondrocyte differentiation. TGFβ_1_ stimulated MSC differentiated into chondrocytes at x20 magnification. MSC were cultured as pellets after detachment from tissue culture plastic and differentiated into chondrocytes.

**References**

1. Holt AP, Haughton EL, Lalor PF, et al. Liver myofibroblasts regulate infiltration and positioning of lymphocytes in human liver. Gastroenterology 2009;136:705-14.

2. Garg A, Houlihan DD, Aldridge V, et al. Non-enzymatic dissociation of human mesenchymal stromal cells improves chemokine-dependent migration and maintains immunosuppressive function. Cytotherapy 2014;16:545-59.

3. Edwards S, Lalor PF, Tuncer C, et al. Vitronectin in human hepatic tumours contributes to the recruitment of lymphocytes in an alpha v beta3-independent manner. Br.J.Cancer 2006;95:1545-1554.
