# Supplementary material for: TGFβ1 priming enhances CXCR3‐mediated mesenchymal stromal cell engraftment to the liver and enhances anti‐inflammatory efficacy
Source: J Cell Mol Med. 2023 Feb 23;27(6):864–78. doi: 10.1111/jcmm.17698 (PMC10002976; doi:10.1111/jcmm.17698)
Supplement: Supplementary file 2 — Appendix S1. [file JCMM-27-864-s001.docx]

**Human liver tissue and cell culture**

Human liver Tissue used in this study was from patients at the Queen Elizabeth Hospital Birmingham, UK. Normal tissue was surplus to transplantation or from tumour margin samples. Diseased tissue was also obtained during transplantation for end-stage disease (Primary Biliary Cirrhosis (PBC), Primary Sclerosing Cholangitis (PSC), Autoimmune hepatitis (AIH), Non-alcoholic steatohepatitis (NASH) and Alcoholic Liver Disease (ALD)). All samples were collected with local research ethics committee approval (reference number 06/Q2702/61) and informed, written patient consent.

Human Mesenchymal Stem cells (hMSC) from healthy donors were purchased from Lonza Group Ltd, (MSC: Lonza Poietics®) and cultured in human MSC Growth Medium (hGM) according to manufacturer’s instructions. Cells were designated passage 1 upon arrival and CD45Cells were detached from culture flasks using non-enzymatic methods as previously described. After detachment cells were washed with PBS supplemented with 1% foetal calf serum (FCS, Invitrogen, UK)) and centrifuged for 5 minutes at 900g before resuspension in hGM. Tri-lineage differentiation potential was confirmed as previously described. Where indicated, MSC were stimulated with predetermined optimal concentrations of cytokines (TGFβ_1_, 5ng/ml, IL-4 10ng/ml or IL-10 50ng/ml, all from Peprotech UK) or media alone, for 10 minutes to 24 hours in hGM at 37°C (supplementary figure 4). Supernatants were collected from these cells after 24 hours treatment and used for quantification of secreted proteins using a human proteome profiler array kit (ARY007 R+D systems UK) according to manufacturer’s instructions. Fixed permeabilised cells were also stained in situ for chemokine expression using optimal concentrations of primary antibody (10-50ug/ml CCR9 or CXCR7 (Pierce) CXCR3 or CCR5 (Abcam) and CXCR7 (R+D systems)) or isotype and species matched controls in an indirect-HRP protocol with Nova Red substrate (Vector labs).

**Adhesion and migration assays**

Adhesion of MSC to cultured cell monolayers, human liver tissue sections or mouse liver sections (control and CCl_4_ treated) was assessed using a modified Stamper Woodruff static adhesion assay. Cultured MSC were detached, washed and incubated with 1µM CFSE per ml for 10 minutes at 37°C. MSC were washed thoroughly to remove unincorporated CFSE and re-suspended in PBS 0.1% BSA (Sigma) to 1x10^6^/ml. CFSE-labelled MSC were added to thawed tissue sections or cultured cells wells in PBS and incubated for 60 minutes at room temperature. Substrates were then washed with PBS and fixed for 5 minutes with methanol prior to washing to remove unbound MSC. Microscopic images of cell monolayer adherent CFSE-labelled MSC were taken at x40 magnification using an inverted fluorescence microscope. Image J threshold analysis was performed on these images to calculate the % of field area pixels which were fluorescent. Alternatively the total number of CFSE labelled MSC bound to tissue sections was counted in 30 fields of view (fov) at x40 magnification in the absence or presence of function blocking antibodies (anti-human CCR5, or CXCR3 both 20ug/ml from R+D systems). Image J analysis was also used to calculate % area of less differentiated necrotic tissue in CCl_4_ injured murine tissues stained using haematoxylin and eosin according to standard protocols. 10 fov from 3 sections from each animal were imaged at 40x magnification for analysis.

To assess migration of control or TGFβ1-stimulated MSC we used a modified 48 well Boyden chamber as previously described[^3^](#_ENREF_3). MSC chemotaxis was determined in a modified 48-well Boyden chamber. Pre-determined optimal concentrations of chemokines (0-500ng/ml CCL17, CCL22, CCL4, CCL5, CCL8, CXCL10 and CXCL11 – all from Peprotech UK) in serum-free media were placed in the lower wells. A polycarbonate membrane with 8µm pores (Neuroprobe) separated MSC (control or pre-stimulated with TGFβ_1_) in the upper wells from the chemoattractants. After incubation at 37oC for 24 hours, filters were removed, air-dried and stained using Diffquick (Medion Diagnostics). Migrated MSC on the lower face of the membrane were counted in 5 fields of view (x40 magnification) per well using a bright-field microscope. Data are expressed as average migration relative to movement towards media alone.

**T cell proliferation assays.**

Ability of TGFβ_1_stimulated MSC to inhibit proliferation of co-cultured activated CD3^+^CD4^+^CD25^-^ T effector cell was determined flow cytometrically using the MSC Suppression Inspector kit (Miltenyi Biotec) according to manufacturer’s instructions. T cells and MSC were incubated at ratios of 1:80 to 1:5 MSC to T cells. MSC were untreated or had been pre-stimulated with 5ng/ml TGFβ_1_ in the presence or absence of 50µM indomethacin for 24 hours. Numbers of CFSE positive T effector cells were quantified after 24 hours and expressed as number of CD3^+^CD4^+^CD25^-^ T effector cells per µL.

**Flow cytometry**

Cell surface or intracellular chemokine expression was assessed using flow cytometry. Non-enzymatically detached control or cytokine stimulated MSC were washed and re-suspended in FACS buffer (PBS + 1% FCS), counted and diluted to 1x10^6^ cells/ml prior to labelling with a live/dead marker (APC Live/dead Kit, Invitrogen) according to manufacturer’s instructions. Labelled cells were washed twice with FACS buffer and re-suspended to 1x10^5^ cells per tube and for intracellular staining, cells were permeablised using the BD Cytofix Fixation/Permeablisation kit (BD Biosciences) according to manufacturer’s instructions. Cells were then washed and labelled with primary antibody or isotype and species matched controls (all 1/10 manufacturers stock from R+D Systems, UK) and incubated for 30 minutes at 4°C in the dark. Cells were washed, labelled with secondary antibody (Polyclonal Goat anti-Mouse PE conjugate, R+D systems UK, 1/10 manufacturers stock) and then re-suspended in 500µl FACS buffer. Labelled cells were analysed using Dako Cyan ADP flow cytometer and Summit version 4.3 software(Dako). Data are expressed as percentage positive cells or median fluorescent intensity of live cells compared to isotype matched controls[^2^](#_ENREF_2).

**qPCR**

Analysis of expression of chemokine receptor mRNA by control or cytokine stimulated MSC was performed using commercially available qPCR kits (Roche) on the Roche Lightcycler 480 instrument. cDNA was tested for the chemokine receptors CCR4, CCR5, CCR9, CXCR3, CXCR4 and CXCR7 using primers designed by the Roche primer design library (Roche probe IDs 25, 14 and 63, 25 and 56, 58 and 79, 47 and 36 respectively). The reaction mix was composed of 1µl of the forward primer and 1µl of the reverse primer, 0.4µl of the probe, 5.6µl RNase free H2O and 10µl of probe master (Roche QPCR Kit), which was added to 2µl cDNA to give a total reaction volume of 20µl in a well of a Roche LC 480 Multi-well, 96 well plate alongside a positive control sample (cDNA from PBMC). β Actin was used as a housekeeping gene (probe ID 58) to which the threshold cycle (Ct) values of the target gene were normalised. Multi-well plates were covered with Lightcycler 480 sealing film (Roche) before they were inserted into the Lightcycler 480 QPCR machine. Differential expression levels were calculated according to the 2-ΔΔCt method and the results were analysed using Lightcycler 480 SW 1.5 software.

**Hepatic engraftment of MSC**

All animal procedures were conducted in in accordance with UK laws with the approval of the Home Office and local ethics committees (PPL 40/3201). Blood was collected by cardiac puncture and serum was used to quantify AST, ALT and bilirubin levels in experimental animals in the Clinical Biochemistry Department of Birmingham Womens Hospital.

Carbon tetrachloride (CCl_4,_ Sigma Aldrich) diluted 1/4 in mineral oil (Sigma) was administered by intraperitoneal (IP) injections (1ml/Kg, acutely as a single injection) into 9 week old C57Bl/6 wild type mice ( supplementary figure 3). After CCl_4_ administration, mice were either sacrificed and livers collected for sectioning, or mice were used for portal vein infusion experiments. Here cytokine-stimulated or untreated CFSE-labelled MSC (5x10^6^ cells/ml in PBS containing 0.1% BSA) were infused into the portal vein of uninjured or injured (acute single dose CCl_4_ treatment) anaesthetised mice. Where indicated, MSC were pre-incubated with blocking antibodies (anti human CXCR3, CCR5 or CXCR4 at 20ug/ml, all from R+D systems) raised against chemokine receptors for 15 minutes at 37°C, washed and re-suspended in PBS 0.1% BSA. Post injection, bleeding was encouraged to clot using Spongostan (Ethicon, National Veterinary Services). Mice were maintained under anaesthesia for 15 minutes to allow engraftment of infused cells. Mice were then culled and the livers were immediately snap frozen. Serial sections were cut from all lobes of the liver and CFSE-labelled cells were counted by fluorescent microscopy in 10 fields of view on 4 independent sections over 4 depths from each mouse liver. Alternately sections were used for immunohistochemical quantification of CD45^+^ immune cells (5ug/ml rat anti human CD45, R+D systems) using a standard indirect protocol (Vector Immpress).

To study engraftment of MSC in liver and non-hepatic organs, MSC (control or 5ng/ml TGFβ_1_-stimulated) were labelled with Direct red (DiR 5uM, Invitrogen) according to manufacturer’s instructions. 1x10^6^ cells were injected into the tail vein mice that had been acutely injured with CCl_4_ (1ml/kg IP, 72 hours). Organs were harvested 72 hours later and imaged using an IVIS Spectrum Imaging System (Perkin Elmer). Fluorescent and photographic images of individual organs were analysed using Living Image software. Here regions of interest (ROI) were defined around each organ and after subtraction of background signals, the radiance efficiency was calculated. Mean radiant efficiency was calculated for a minimum of 6 mice in each treatment group. In addition organs were non enzymatically digested using a Gentlemacs digestor (Miltenyi) and the absolute number of DiR-labelled MSC was quantified for each organ.

**Liver Digest Analysis**

Liver samples were homogenised by crushing through the 70µm nylon mesh. The single cells were separated by gradient centrifugation using the OptiPrep, and then stained with live/dead dye at 4C for 30min. Non-specific binding was blocked by incubating cells with anti-mouse FcR antibody (CD16/CD32) for 15min at 4C in PBS buffer, and then stained with primary conjugated antibodies. All  antibodies were used at 1:200 dilution. Flow cytometry was performed with Fortessa analyser with FACSDiva6.2 software(BD Biosciences), with data subsequently analysed with Flowjo software (version 10.1). Flow cytometric analysis showing liver macrophages (gated CD45^+^CD3^-^CD11b^+^F4/80^+^Ly-6G^-^), M1-macrophages (gated CD45^+^CD3^-^CD11b^+^F4/80^+^Ly-6G^-^Ly6C^hi^) and M2-macrophages (gated CD45^+^CD3^-^CD11b^+^F4/80^+^Ly-6G^-^Ly6-C^lo^).
